# Elucidation of lipid nanoparticle surface structure in mRNA vaccines

**DOI:** 10.1038/s41598-023-43898-x

**Published:** 2023-10-05

**Authors:** Mingzhang Maple Wang, Caitlin N. Wappelhorst, Erika L. Jensen, Ying-Chih Thomas Chi, Jason C. Rouse, Qin Zou

**Affiliations:** 1 Analytical Research and Development, BioTherapeutics Pharmaceutical Sciences, Pfizer, Inc., 875 Chesterfield Parkway West, Chesterfield, MO 63017 USA; 2 Analytical Research and Development, BioTherapeutics Pharmaceutical Sciences, Pfizer, Inc., 1 Burtt Road, Andover, MA 01810 USA

**Keywords:** Nanoscale biophysics, Gene therapy, Solution-state NMR

## Abstract

Lipid nanoparticles (LNPs) have been used as a carrier for messenger RNA (mRNA) vaccines. Surface properties of LNPs are important to the stability and function of mRNA vaccines. Polyethylene-glycol (PEG) is a functional lipid at the surface of LNPs that improves colloidal stability, increases circulation time, and impacts cellular uptake. In this study, we explore in-depth lipid composition at the surface of mRNA-LNPs using high-field nuclear magnetic resonance (NMR) spectroscopy. Our results provide a unique surface lipid profile of intact LNPs identifying PEG chains and partial ionizable lipids are present with quantification capability. The surface PEG density is determined to reveal the brush-like conformation on the surface of mRNA-LNPs. Furthermore, we implement a diffusion NMR strategy for routine testing of formulated drug products during drug development. Comparative NMR analysis of different vaccine preparations and stability samples provides a global view of the mRNA-LNP surface structure for enhanced product knowledge.

## Introduction

The success of mRNA-based COVID-19 vaccines against SARS-CoV-2 virus has brought attention to lipid nanoparticles (LNPs) as drug delivery vehicles. In addition to the protective role of maintaining mRNA integrity, LNPs are important to cellular uptake and ultimate delivery of mRNA to the cytosol for expression. It is suggested that both LNP size and surface charge affect cellular uptake. As a result, there has been extensive work done to characterize and control LNP size, size distribution, and surface charge by various techniques, such as dynamic light scattering (DLS), sedimentation Field Flow Fractionation (FFF), laser Doppler anemometry, transmission electron microscopy (TEM), and scanning electron microscopy (SEM)^1–3^.

**Figure 1:**
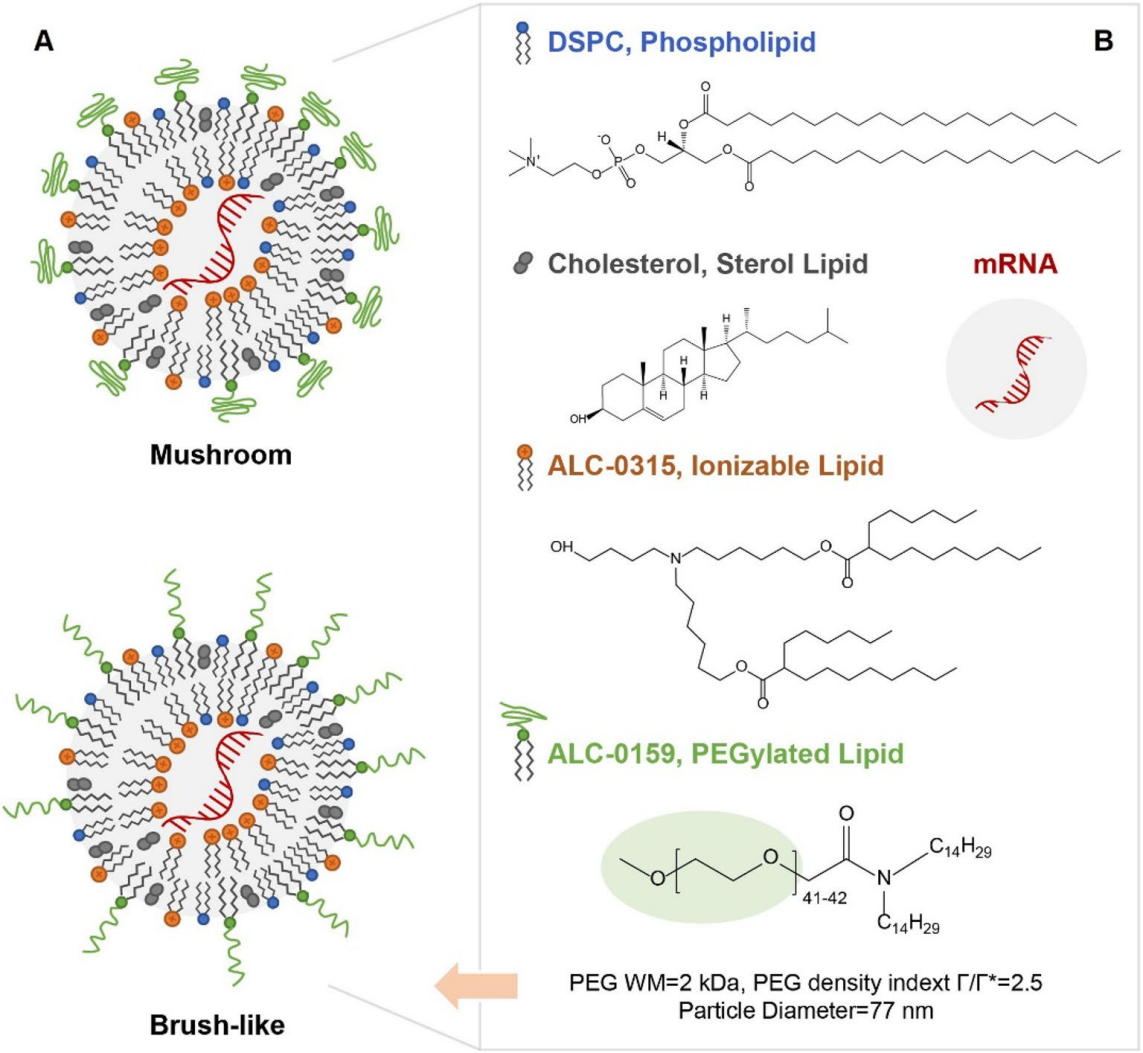
Surface PEG Models and COMIRNATY mRNA Lipid Nanoparticle Composition. (**A**) two primary models of surface PEG conformation: mushroom and brush-like. (**B**) COMIRNATY lipid nanoparticle composition.

The current LNPs used for both Pfizer-BioNTech and Moderna COVID-19 mRNA vaccines contain four different types of lipids: an ionizable lipid, a PEGylated lipid, cholesterol, and a helper lipid^4^. Due to the various lipid compositions and the complexity of the self-assembled particles, the mRNA-LNP structures are still not clear. To date, three models have been proposed, including multilamellar vesicles^5,6^, nanostructured core^7^, and homogeneous core shell^8^. The ionizable lipid, besides interacting with mRNA, is important to adjust the surface charge in controlling the release of mRNA to the cytosol through endosomal escape upon pH shift^9^. Another important surface property is the presence of polyethylene glycol (PEG) as part of the PEGylated lipid. PEG moieties on the LNP surface improve colloidal stability, enhance solubility, extend in vivo circulation time and decrease the immunogenicity of the vaccines for better safety and efficacy. It is reported that the surface PEG density impacts the retention and distribution of biodegradable nanoparticles^10^. PEG conformation is related to the extent of PEG density, and it is modeled either as a mushroom (sparsely packed) or brush (densely packed) format (see Fig. 1A). PEG surface structure has been shown to affect the adsorption of plasma proteins, cellular uptake, in vivo circulation, etc^11,12^. The importance of PEGylation on nanoparticles, for example, some liposomes, is recognized by regulatory agencies as one of the critical physicochemical properties, and the FDA provided guidance that expects sponsor-investigator due diligence with respect to the characterization and understanding of PEG on the surface of such nanoparticles (https://www.regulations.gov/docket/FDA-2017-D-0759).

There are multiple ways to characterize PEG on the surface of nanoparticles, e.g., labeling surface PEG with fluorescent stain or colored compounds, X-ray photoelectron spectroscopy (XPS) and nuclear magnetic resonance (NMR) (see review by Shi et al.^11^). 1D proton NMR is the most straightforward method that provides direct detection and quantification of the surface PEG. For example, Garcia-Fuentes et al.^13^ used 1D proton NMR to evaluate the surface PEG on the lipid nanoparticle composed of tripalmtin, lecithin and PEG-stearate. Xu et al.^10^ studied extensively the surface PEG density and conformation of a mucus-penetrating nanoparticle by NMR. In addition, Retout et al.^14^ used NMR to characterize the thiol-PEG functional group on the surface of gold nanoparticle. NMR has also been used in combination with XPS to characterize and quantify the surface PEG on a polymeric nanoparticle and the results showed that the high level of surface PEG was correlated to the reduced uptake by macrophage^15^. Here, we employed high-field NMR spectroscopy to characterize surface structure of the intact mRNA-LNPs in the COMIRNATY vaccine (see Fig. 1), as well as determine a surface PEG model by measuring the PEG molecular weight and density on the LNP surface. Furthermore, we implemented a 1D ^1^H diffusion NMR sequence as a routine method for characterizing formulated LNP that minimizes the sample manipulation of drug product. Our data on aged LNPs demonstrated that 1D ^1^H high-field NMR spectroscopy can be used to detect the surface lipid changes associated with a perturbed LNP surface structure.

## Lipid nanoparticle surface characterization

COMIRNATY vaccine drug product, composed of mRNA molecules encapsulated by LNPs, was used as a case study for NMR surface characterization. The LNPs contain PEGylated lipids (ALC-0159), ionizable lipids (ALC-0315), phospholipid 1,2-distearoyl-sn-glycero-3-phosphocholine (DSPC) and cholesterol (see Fig. 1B) that are mixed at a defined ratio to facilitate formation of a stable nanostructure. The surface structure of LNP is one of the key physicochemical properties for this novel modality. By design, the surface PEG coating can potentially improve the stability, safety, efficacy, and pharmacokinetic profiles of the mRNA-LNPs by protecting LNP structural integrity and increasing circulation half-life^10–12^.

Before probing the mRNA-LNP surface, the four individual lipid components were characterized in disrupted LNPs to verify the lipid composition. The mRNA-LNPs were disrupted by dissolution in deuterated chloroform. Determination of lipid composition in disrupted LNP by ^1^H NMR has been reported by Garcia-Fuentes and co-workers^13^. Individual lipids were characterized by 1D ^1^H NMR in chloroform as shown in Figure S1 to confirm the peak assignments of the disrupted LNPs; carbon chemical shift assignments are reported for ALC-0159 and ALC-0315 in the supplemental information section (Figures S2 and S3, respectively). Multiple identifiable proton signals in each lipid revealed a good alignment with the signals from the LNP sample, which demonstrated the presence of the four expected lipids in the disrupted LNP. Total lipid content of the LNP was further quantified using unique protons from the four lipids that were well-resolved. The unique proton signals of the individual lipids in the disrupted LNP spectrum were integrated and ratioed with signals of internal standard (TMS) as reference. The abundance of all four lipids determined by NMR is listed in Table S1, which verifies the presence of four individual lipids at the expected abundance in the LNP according to the COMIRNATY product insert (please note that lipid quantitation is determined by validated methods in a GMP quality control laboratory environment; NMR is performed as a non-GMP method for heightened characterization of various product quality attributes and it is not used for mRNA-LNP lot release and stability).

**Figure 2:**
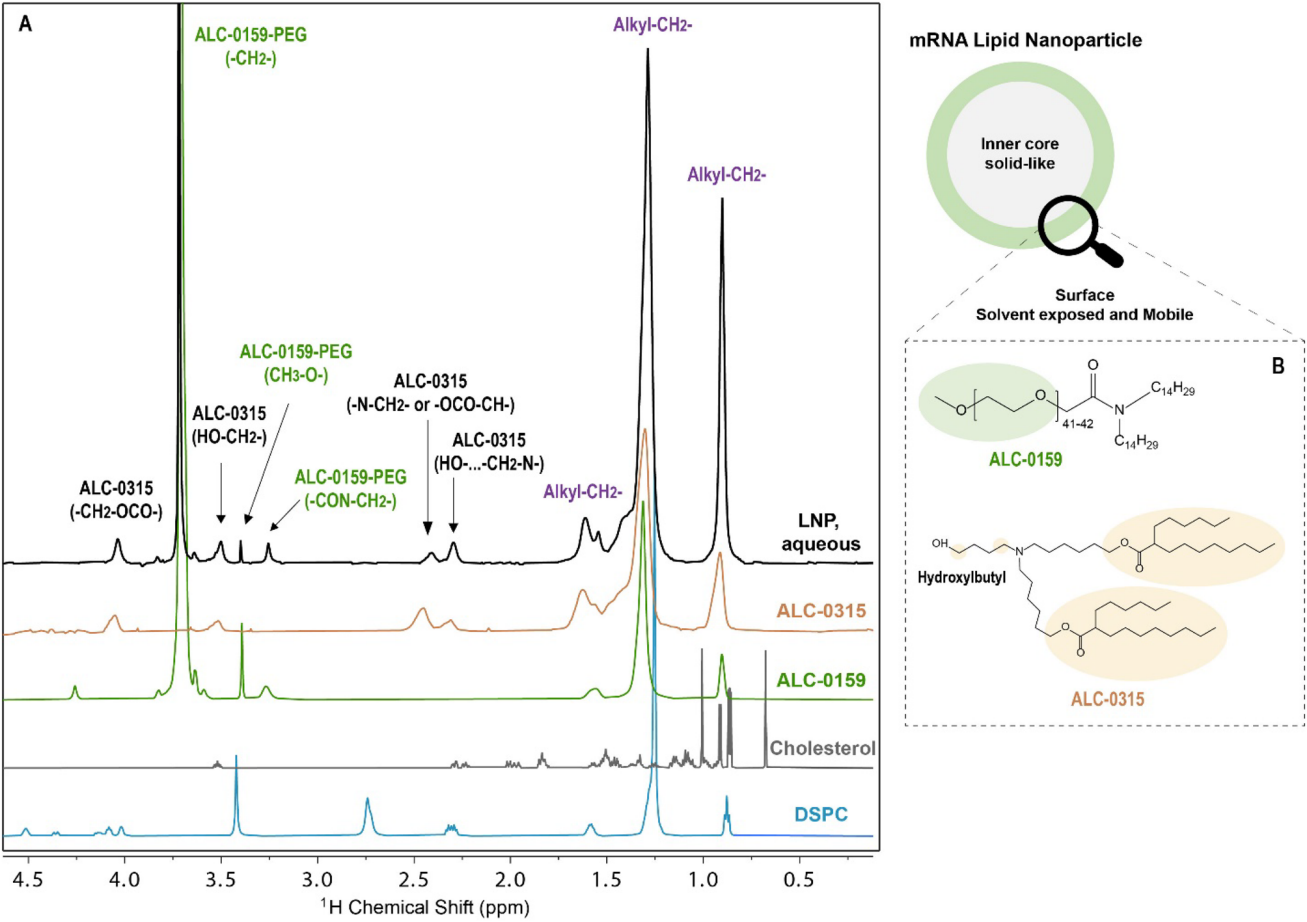
Surface Characterization of Intact mRNA-LNPs by 1D ^1^H NMR. (**A**) top spectrum: intact mRNA-LNPs in aqueous phosphate buffer; Bottom four spectra: individual lipids: ALC-0315 (aqueous, PBS), ALC-0159 (aqueous, PBS), cholesterol (chloroform), and DSPC (chloroform). Proton signals in the mRNA-LNP spectrum were labeled in green for ALC-0159, and in black for ALC-0315; overlapped signals from both lipids were labeled in purple. (**B**) the detected surface protons were annotated in the molecular structures of ALC-0159 and ALC-0315. Spectra are not at equivalent scale with the lipid concentration.

In solution state NMR spectroscopy of particle suspensions, two relaxation parameters spin–lattice, longitudinal relaxation (T_1_) and spin–spin, transverse relaxation (T_2_) are significantly impacting the intensity and lineshape of resonance peaks. The molecular components present on the solvated surface with sufficient mobility (T_1_≈T_2_ at the same time scale) will give rise to detectable or well resolved resonance peaks. On the other hand, the internal core (solid-like) of LNP is tightly packed with aliphatic groups with much lower mobility (very short T_2_) that is “invisible” in the NMR analysis^16,17^. Benefiting from this property, the flexible surface structure of intact LNPs in aqueous solution can be characterized by 1D ^1^H NMR. As shown in Fig. 2, the proton signals representing PEG in ALC-0159 were observed in abundance on the surface of LNP, in addition to protons representing partial ALC-0315 structure. The unique ALC-0159 methoxide protons (^1^H 3.38 ppm, peak width 4 Hz) at the PEG terminus and the repeating methylene protons (^1^H 3.71 ppm, peak width 6 Hz) were well resolved, which indicates that the PEG chains are present on the LNP surface and showing high mobility. The methylene next to secondary amine (C1 proton of N-alkyl chain) in ALC-0159 (^1^H 3.25 ppm, peak width 21 Hz), the methylene next to tertiary amine in the ALC-0315 (^1^H 2.4–2.3 ppm, peak width 30–60 Hz), ɑ-ester proton in the ALC-0315 (^1^H 4.15 ppm, 30 Hz), as well as alkyl chains (^1^H 1.6–0.9 ppm, peak width 20–60 Hz) in both lipids were observed in the intact LNP. These peaks show significantly more broadening than signals from PEG chains that indicates these chemical groups are likely close to surface but have relatively less mobility on the LNP surface. The peak broadening is primarily impacted by T_2_, and both properties can be used to evaluate the relative mobility of each surface lipid group. (See Table S2). DSPC and cholesterol were not observed on and/or close to the surface, suggesting that they are much less mobile and tightly associated within the LNP as structural lipids.

Moreover, the ALC-0315 methylene groups (^1^H 2.4–2.3, 4.15 ppm), hydroxylbutyl group (3.51 ppm) and alkyl chains (1.6–0.9 ppm) show much lower peak area comparing with the total lipid analysis data in disrupted LNP analysis (see Figure S4, equivalent scale with the lipid concentration). The total quantity of individual lipids has been determined by disrupting the LNP in chloroform. The lower peak area of surface ALC-315 peaks suggests only partial ALC-0315 lipids are close to the LNP surface. The surface PEG chains and hydroxylbutyl group of ALC-0315 were quantified in the intact LNP with aqueous buffer by NMR. Therefore, the relative abundance can be determined for surface PEG chains and ALC-0315 that is on and/or close to the surface, as shown in Table S1. A high PEG coating efficiency at about 90% was demonstrated by comparing the amount of PEG on the surface with respect to the total amount of PEG in the particle. Approximately 59% of ALC-0315 hydroxylbutyl group were detected on the LNP surface likely as a contributor to modulate the respective surface charge. Our data demonstrated the presence of surface PEG corroborate the claim of the purpose on using the PEGylated lipid, as well as provide the evidence of the ionizable lipid on the LNP surface that is playing its dual roles for LNP surface charge regulation and RNA capturing^9^.

## Determination of the mRNA-LNP surface PEG model

To further understand the role of surface PEG in the mRNA-LNPs, an in-depth characterization of surface PEG configuration was performed by using NMR spectroscopy and other biophysical techniques. The surface PEG model can be determined by calculating the surface PEG density, which requires the average molecular weight (MW) of the PEG chains and evidence of spherical morphology for the mRNA-LNPs.

ALC-0159 is a PEGylated lipid consisting of a PEG group (~ 2 kDa) conjugated to a lipid anchor with two 14-carbon saturated alkyl chains. The average MW of the PEG is one of the factors that potentially impact efficacy, circulation time and immune cell uptake^4^. We initially employed NMR spectroscopy to characterize the structure of ALC-0159 lipid and determine the PEG average MW. The 1D ^1^H NMR spectrum of a representative ALC-0159 lot was collected and analyzed by assigning chemical shifts and integrating peak area, as shown in Figure S5. The ^1^H chemical shift assignments were elucidated based on the various functional groups and chains in ALC-0159, and each proton was quantitatively determined to be consistent with the theoretical proton number in the lipid structure by comparing the ratio of peak integrals. Furthermore, the number of PEG repeat units (N) was evaluated by integrating the proton NMR signals in five chosen regions to calculate the corresponding N values based on the lipid structure (See Tables S3–S4 for calculation of N value). The averaged N value determined by NMR can be used to calculate the average MW of PEG. This representative ALC-0159 lot contains an average of 41.5 ± 0.3 PEG chain repeat units, which results in the expected MW of the PEG group of ~ 2 kDa. This is consistent with mass spectrometry results, which is based on the MW distribution of the intact ALC-0159 (data is not shown). NMR provides more accurate weight-average MW values in the case that there is a polymer with large polydispersity present.

The surface PEG density can be evaluated using the model and equations reported by Xu and co-workers^10,18^. Equations are listed in SI section. PEG density, [Γ] was calculated as the number of PEG molecules per 100 nm^2^ surface area on LNP. It was determined by surface PEG moles (M_PEG_, mole), total mass of nanoparticles (W_NP_, g), the density of nanoparticles (g/mL), and the particle diameter (D, nm). Using COMIRNATY as a case study, the average particle diameter was measured as 77 nm by DLS. The spherical shape of mRNA-LNPs was confirmed by asymmetric flow field flow fractionation (AF4) as well as cryogenic electron microscopy (cryo-EM). The density of mRNA-LNPs was determined as 1.0 g/mL (See SI for other techniques). The full surface coverage [Γ*] is equal to 11.0, which indicates the number of unconstrained PEG molecules that occupy 100 nm^2^ surface, was determined from the average MW of the full PEG chain^19^. The PEG density [Γ] (#PEG/100 nm^2^) is 27.3. [Γ/Γ*] is an index to assess the PEG density and conformation on the nanoparticle surface. The ratio [Γ/Γ*] of the COMIRNATY mRNA LNP was 2.5, revealing that a dense PEG brush-like conformation (Fig. 1) has been formed on the surface of the LNP. This type of brush conformation has been reported to form a thicker hydrophilic barrier to protect LNP that is reducing nonspecific protein adsorption and macrophage uptake. Brush-like model is more favorable for stealthy effect of nanocarriers with lower interactions to the cell^18,20–22^.

## Diffusion NMR for routine testing of formulated drug product

**Figure 3:**
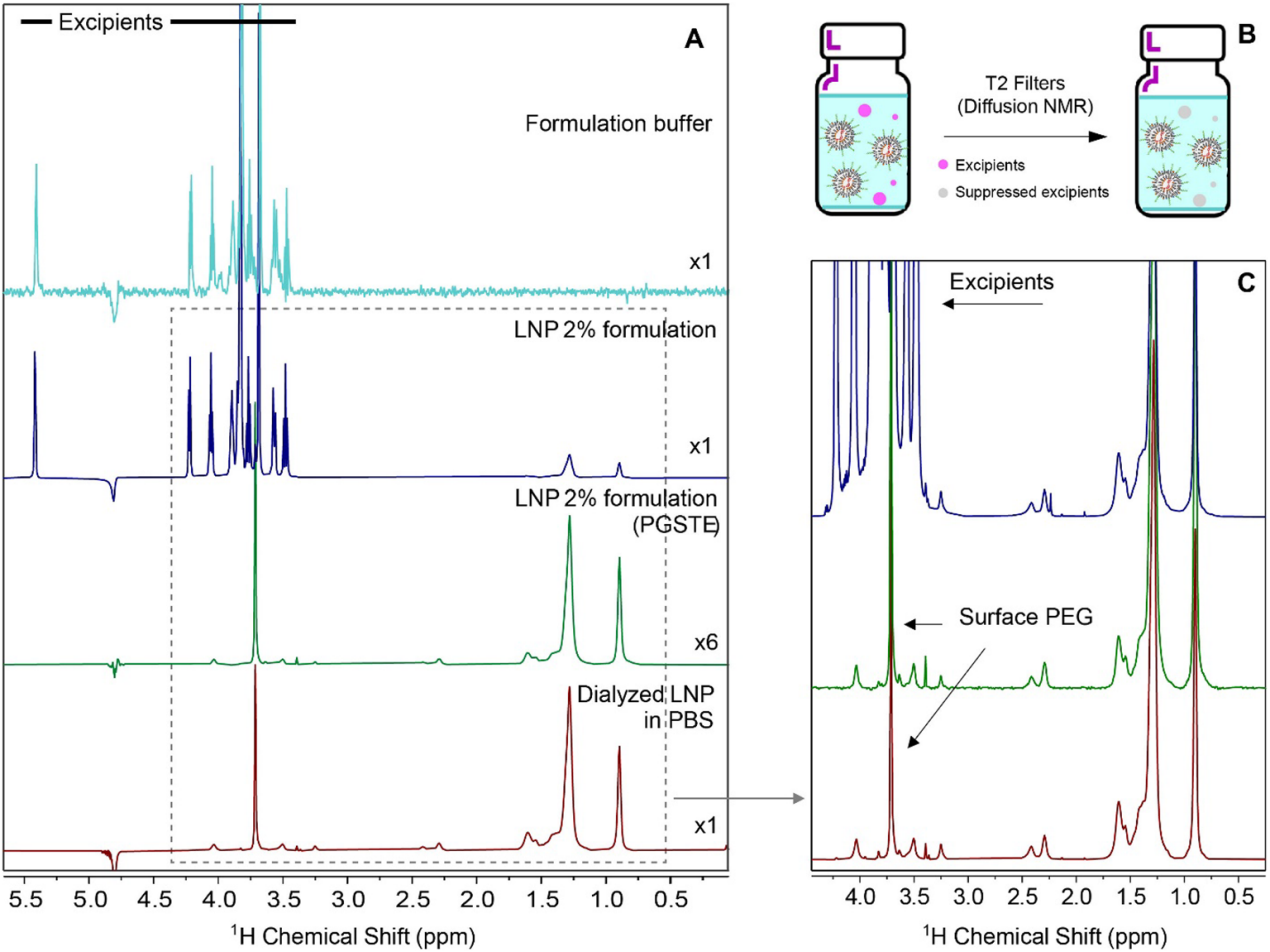
1D ^1^H NMR Spectra of mRNA-LNPs in PBS and a 2% Formulation (post-dilution with PBS). (**A**) top spectrum: the formulation buffer control; Middle two spectra: LNP sample in 2% formulation buffer with and without PGSTE; Bottom spectrum: the dialyzed LNP sample in PBS (bottom). (**B**) a schematic illustrating excipient (pink spheres) signals in formulated drug products are suppressed by T_2_ filters used in PGSTE. (**C**) the expansion of spectra in panel A.

In the process of pharmaceutical characterization and analysis, limited sample manipulation is a best practice to preserve sample integrity as well as increase testing efficiency. For COMIRNATY mRNA-LNP samples, sucrose and other excipients in formulation were preventing good resolution of the surface signal in the intact LNP. Therefore, a diffusion experiment using Pulse Gradient Stimulated Echo (PGSTE)-bipolar gradients^23,24^ was introduced to produce a cleaner spectrum by suppressing the excipient signals, particularly the intense sucrose signals in the formulated LNP samples. Simulated echo-based sequences have been demonstrated to be one of the favorable sequences for efficient solvent suppression with no phase distortions on liposome and monoclonal antibody samples^25–27^. As shown in Figure 3, by comparing the spectra of the dialyzed LNP in PBS and that of the 2% formulated LNP, the surface ^1^H profiles are identical despite the expected lower sensitivity obtained in PGSTE. The large signals of excipients in the range of 4.3–3.3 ppm were significantly suppressed, and the PEG-methylene chains were distinctly detected in the spectrum as the primary components on the surface of LNPs. The repeatability was evaluated by collecting triplicate measurements on a sample and preparing three different samples, respectively (Figure S6). The spectra were quantitatively analyzed by spectral classification tool in the chemometric software package TQ Analyst 9 (Thermo Scientific)^28^ to calculate similarity scores between spectra of different measurements and preparations^29^. The high similarity scores (> 95%) indicate the appreciable repeatability in the 1D ^1^H NMR method for the surface structural characterization of LNP.

**Figure 4:**
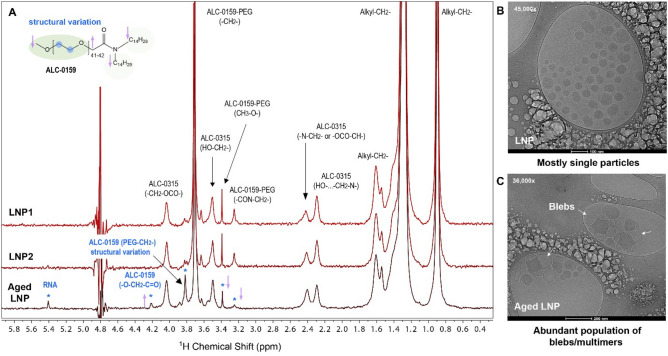
Surface Structural Characterization of Aged LNP by Diffusion 1D ^1^H NMR and cryo-EM. (**A**) 1D ^1^H NMR of two representative mRNA-LNP lots (stored at −80 °C) and one aged mRNA-LNP sample (−20 °C for 3 months). The increased intensity at ALC-0159 (PEG-CH_2_-) peak at 3.8 ppm (bold label) is determined as the methylene with conformation variance that is not identical to that in the intact LNP. (**B**), (**C**) cryo-EM images of a representative mRNA-LNP and the aged LNP sample.

With the cleaner spectrum by suppressing the excipient signals with diffusion pulses, the 1D ^1^H diffusion NMR method is sensitive to monitor potential changes in formulated LNP drug product samples for routine testing in the drug development, stability and manufacture. In Fig. 4, two representative LNP samples (LNP1 and LNP2) were compared with one aged LNP lot that was stored at −20 °C for 3 months. The three samples were from three individual LNP manufacturing runs in PBS/Sucrose formulation. The identical 1D ^1^H profiles from LNP1 and LNP2 indicate that the two lots are highly comparable. Interestingly, several proton signals that are associated with ALC-0159 produced altered peak intensity in the aged LNP lot. Likewise, there is a new peak at 5.4 ppm that appears to correlate with an exposed RNA sugar moiety H1’ signal (see Fig. S7)^30^. The original PEG-methylene signal in the intact LNPs is the large sharp peak at 3.71 ppm. However, the increasing PEG-methylene peak at 3.81 ppm in aged LNPs is discernible and it appears associated with a structural variation of surface PEG at −20 °C for 3 months. The attenuated proton signals of ALC-0159 methoxide at the PEG terminus (^1^H 3.38 ppm) and methylene on C1 of N-alkyl chain (^1^H 3.25 ppm) are accompanied by the growing proton signal of H_ɑ_ between PEG and N-alkyl chains (^1^H 4.19 ppm). These peak intensity changes indicate a small part of ALC-0159 undergoes orientational movement that is leading to PEG chain conformational changes on the surface. Moreover, these types of surface structural changes detected by NMR likely correlate with elevated bleb formation on the aged LNP reported by cryo-EM (Fig. 4C) when compared to representative mRNA-LNPs in Fig. 4B. The subtle difference in the surface PEGylated lipids may be the resulted from small lipid rearrangement of LNP after long storage at -20 °C. The fact that freezing a phosphate buffer at −20 °C results in a pH decrease from pH 7 to pH 3.5^31^, likely is one of the triggers to alter the surface lipid interactions. The LNP surface structural change could impact both colloidal stability and potentially the cellular uptake of the LNP. The significance of the subtle profile change remains under investigation.

## Conclusions

As drug delivery vehicles, the surface properties of LNPs are critical to the stability and function of mRNA vaccines. PEGylated lipid at the surface of LNPs is a particular functional lipid to improve colloidal stability, increases circulation time, and impacts cellular uptake. Our results demonstrate that 1D ^1^H NMR spectroscopy is an essential technique for characterizing the structural features of mRNA-LNPs comprehensively and rapidly. First, NMR can characterize the individual lipids used to fabricate the LNPs; here, the molecular structures and the molecular weight of the PEG chain in ALC-0159 were determined. NMR provides the unique capability to profile the components on the surface of the LNP, in addition to providing confirmatory analysis of the individual lipid components in disrupted LNPs. For COMIRNATY mRNA-LNPs, NMR detected PEG chains on the surface as expected for improving the particle stability and vaccine function, and partial ALC-315 ionizable lipids close to the surface of the LNP for regulating surface charge. PEG density can be accurately determined using quantitative proton NMR data, which revealed LNPs exhibit a dense PEG brush-like conformation on the surface. Benefiting from high sensitivity and resolution at high magnetic field strength, 1D ^1^H diffusion experiment has been used to rapidly assess the formulated LNP surface characteristics in representative COVID-19 vaccine lots, with minimal sample preparation/manipulation during drug developments. NMR is a sensitive analytical tool that is capable of assessing LNP stability by detecting the surface property changes after extended storage. Taken together, our work has provided key structural information for the novel mRNA-LNP modality that contributed immensely to product quality understanding as well as development of the manufacturing process and stable formulations.

## Methods

### NMR analysis of lipid components in the disrupted lipid nanoparticle

To prepare the disrupted samples, mRNA-LNPs were dialyzed to remove sucrose and excipients, followed by speed-vacuum drying. The dried LNPs were resuspended in chloroform-d containing 0.03% (v/v) tetramethylsilane (TMS). Individual lipids, ALC-0159, ALC-0315, distearoylphosphatidylcholine (DSPC), and cholesterol were dissolved in chloroform-d with 0.03% (v/v) TMS at 10–20 mg/mL. Standard 1D ^1^H 90° pulse (zg) with 30 s relaxation delay was performed on each individual lipid and the disrupted LNP in chloroform to characterize the total components. Additional 1D ^13^C spectra were collected on ALC-0315 and ALC-0159 for determining carbon assignments (See Figs. S2–S3). The spectra of individual free lipids were compared with the spectrum of disrupted LNP in chloroform. The unique proton signals from each lipid were observed in the disrupted LNP spectrum confirming the presence of four different lipids in the LNP (see Fig. S1). The well-resolved lipid signals were used to calculate the lipid abundance in the LNP by qNMR^32^ utilizing the peak integrals of lipids and the internal reference TMS. Additionally, a careful peak deconvolution was performed using data process software MestreNova 14.1 to minimize the potential overlapping impact between lipids. Note in general, the qNMR method precision is within 5%^32^.

### NMR analysis of surface structure of intact LNPs

To fully characterize the surface structure of the intact LNP and calculate the PEG density, the formulated COMIRNATY mRNA vaccine was dialyzed into 0.2 × Dulbecco's phosphate-buffered saline (DPBS), pH 7.4, removing sucrose and other excipients. The LNP sample for NMR was prepared in 10% D_2_O containing 0.005 wt.% 3-(trimethylsilyl) propionic-2,2,3,3-d4 acid, sodium salt (TSP) for chemical shift calibration. LNP NMR spectra were recorded on a Bruker NEO 800 MHz spectrometer, equipped with a 5 mm proton-optimized triple resonance NMR inverse (TCI) cryoprobe at 25 °C (298 K).

The PEGylated lipid, ALC-0159, and the ionizable lipid, ALC-0315, were dissolved in 0.2 × DPBS, pH 7.4 aqueous buffer at 1–2 mg/mL for collecting lipid reference spectra. Lipid NMR spectra were collected by a Bruker NEO 600 MHz spectrometer, equipped with a 5 mm double resonance broad band ^1^H/^19^F (BBFO) cryoprobe at 25 °C (298 K). All spectra were processed and analyzed using MestreNova 14.1.

### NMR analysis of formulated LNPs

To investigate diffusion sequence for suppressing excipient signals in the formulated LNP, the COMIRNATY mRNA vaccine was diluted with 0.2xDPBS with the final sample containing 2–5% formulation buffer. Standard 1D ^1^H 90° with water suppression using excitation sculpting (zgesgp), and 1D ^1^H pulse field gradient stimulated echo (PGSTE, stebpesgp1s1d)^23^ were performed for characterizing the surface structure of the intact LNP. For 1D excitation sculpting experiment, the acquisition time and relaxation delay were 2.6 s and 30 s, respectively. For diffusion experiment, the acquisition time and relaxation delay were 1.3 s and 2.5 s, respectively. The diffusion time was 60 ms, the duration of gradients was 2 ms, and the Z gradients were applied at maximum strength (56 G/cm) at 18.8 Tesla magnetic field (800 MHz).

### Supplementary Information

Supplementary Information.

## Data Availability

The data generated or analysed during this study are included in this published article (and its Supplementary Information files).
