# Supplementary material for: Elucidation of lipid nanoparticle surface structure in mRNA vaccines
Source: Sci Rep. 2023 Oct 5;13:16744. doi: 10.1038/s41598-023-43898-x (PMC10556076; doi:10.1038/s41598-023-43898-x)
Supplement: Supplementary file 1 — Supplementary Information. [file 41598_2023_43898_MOESM1_ESM.pdf]

Supplementary Information

Elucidation of Lipid Nanoparticle Surface Structure in mRNA Vaccines

Mingzhang Maple Wang[1*], Caitlin N. Wappelhorst[1], Erika L. Jensen[1],
Ying-Chih Thomas Chi[1], Jason C. Rouse[2], Qin Zou[1*]

[1] Analytical Research and Development, BioTherapeutics Pharmaceutical Sciences, Pfizer, Inc., 875 Chesterfield Parkway West, Chesterfield, MO 63017, United States
[2] Analytical Research and Development, BioTherapeutics Pharmaceutical Sciences, Pfizer, Inc., 1 Burtt Road, Andover, MA 01810, United States

*Corresponding authors

Lipid Component Analysis in the Disrupted Lipid Nanoparticle

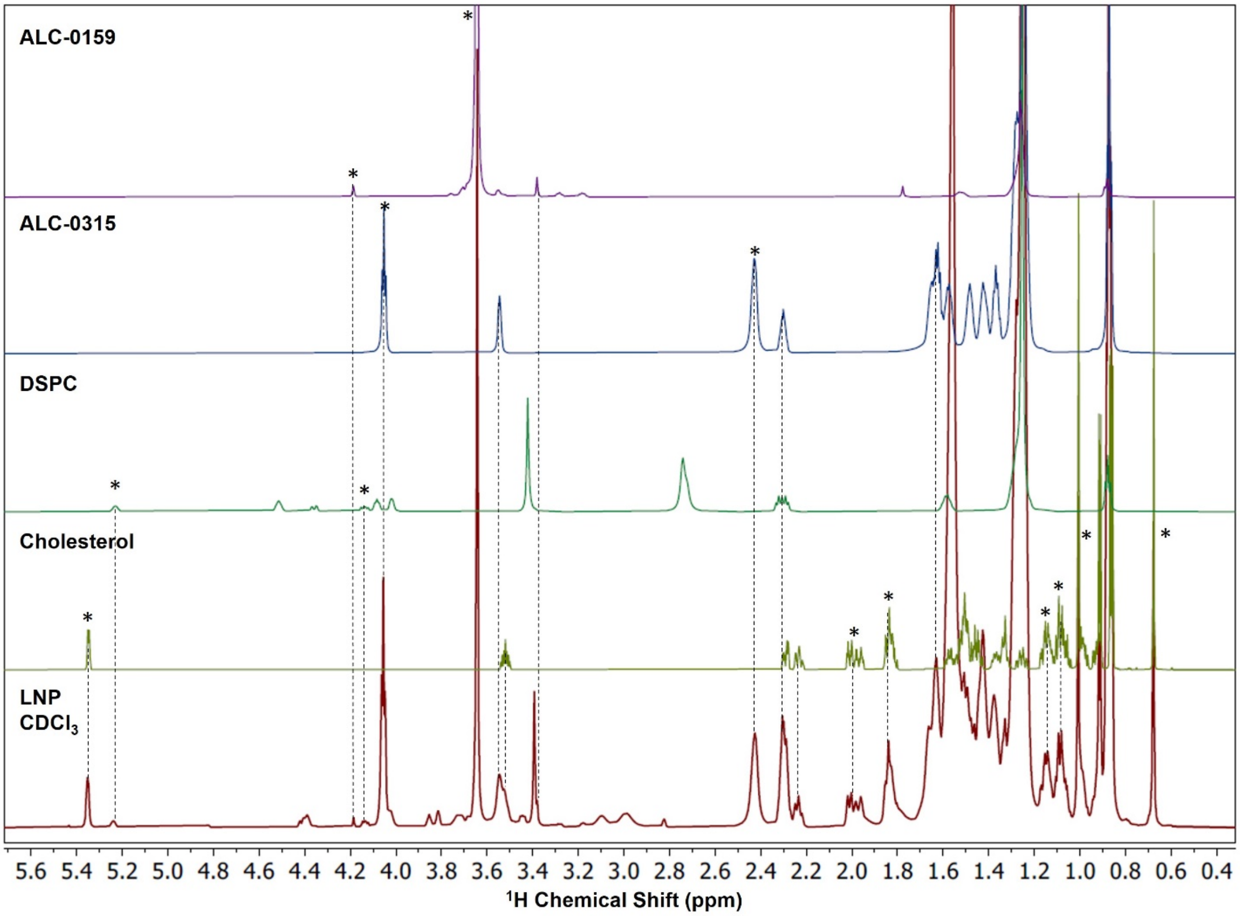

Figure S1. 1D ^{1}H NMR Spectra of Individual Lipid and Disrupted LNP in Chloroform. Unique protons (*) from each individual lipid were used to quantify total lipid components in LNP.

^{13}C NMR Spectra of Lipids: ALC-0315 and ALC-0159

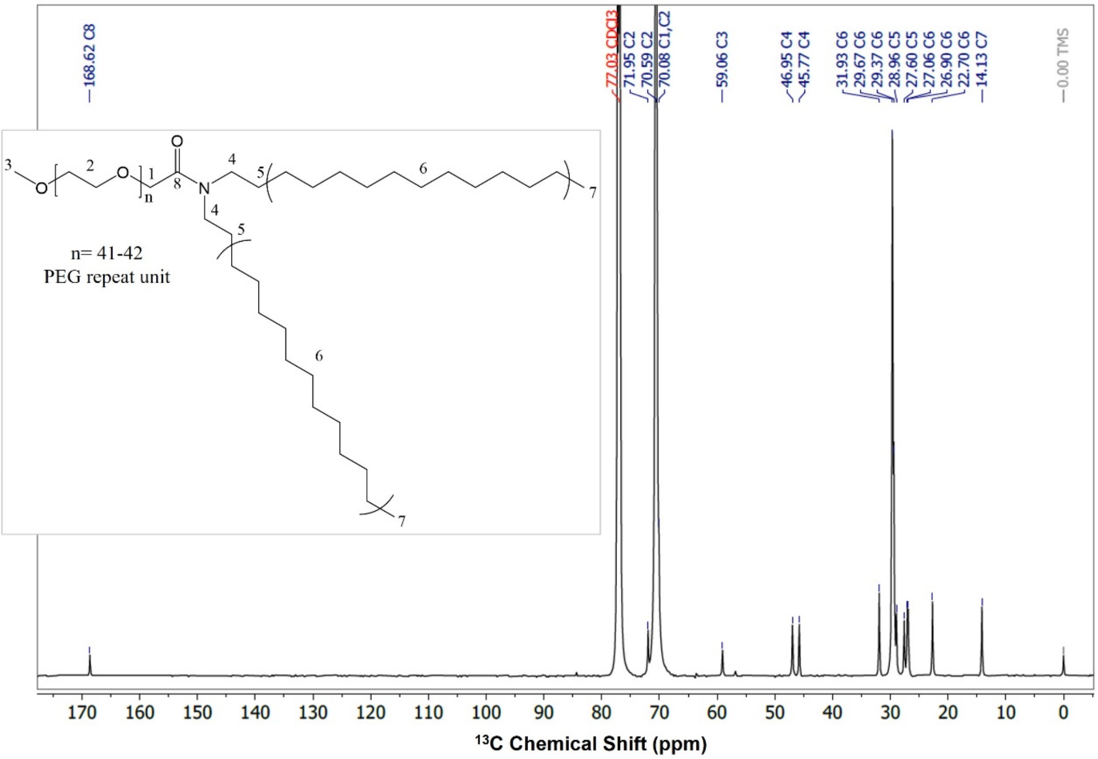

Figure S2. 1D ^{13}C Spectrum of ALC-0159 for Determining Carbon Assignments.

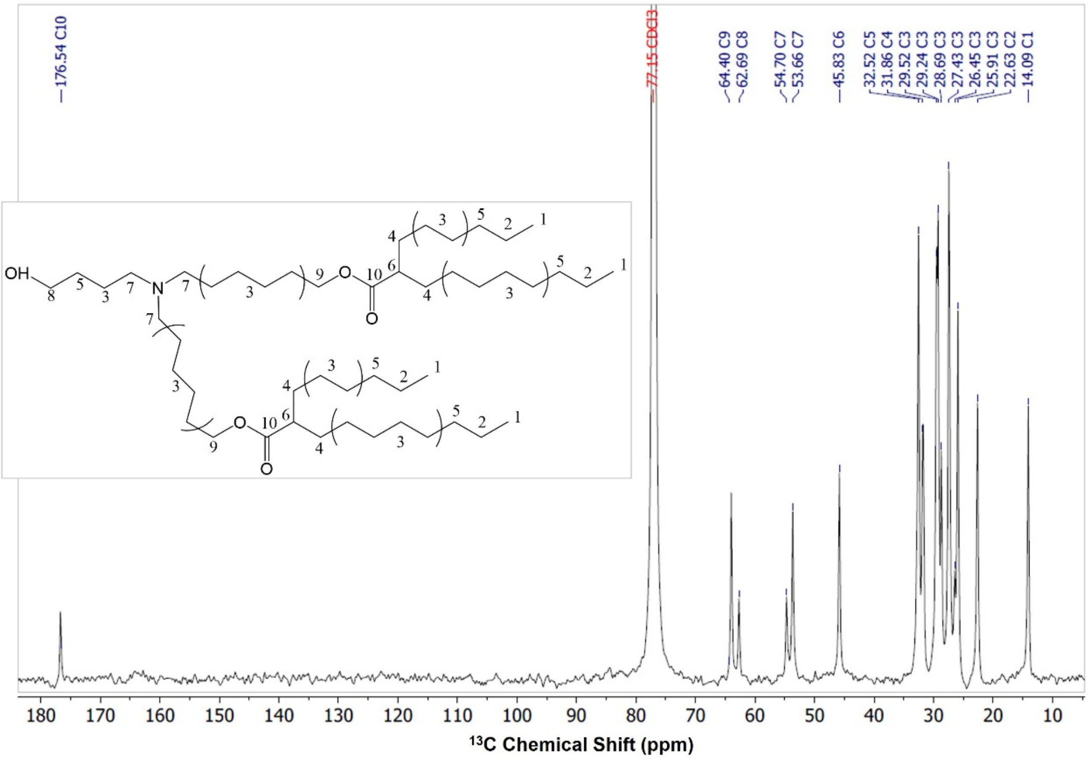

Figure S3. 1D ^{13}C Spectrum of ALC-0315 for Determining Carbon Assignments.

Lipid Nanoparticle Surface Characterization

Table S1. Relative Lipid Abundances Detected by NMR

Lipid	Molar ratio (%) in the Disrupted LNP [a]	Well-resolved Functional Group on the Surface	Molar ratio (%) Detected on the LNP Surface (surface/individual lipid)
ALC-0315	45.5 ± 1.0%	Hydroxylbutyl group	59%
ALC-0159 [b]	2.1 ± 0.3 %	Surface PEG (methoxy and methylene)	90%
DSPC	9.5 ± 0.5%	Not detected	N/A
Cholesterol	42.9 ± 0.1%	Not detected	N/A

[a] qNMR method precision is within 5%. Please note NMR is performed as a non-GMP method for heightened characterization of various product quality attributes.
[b] ALC-0159 abundance is < 2% according to COMIRNATY product insert. The larger deviation is due to the limited method precision at low concentration species.

Table S2. Transverse (T_2) Relaxation Time and Peak Width (Hz) of Surface Lipid in the Intact LNPs

	Surface lipid proton	T_2 (s)	Peak width
ALC-0315	Hydroxylbutyl-methylene (3.51 ppm)	0.84	29 Hz
	Tertiary amine-methylene (2.4-2.3 ppm)	0.20	30-60 Hz
	Alkyl-methylene (1.6-1.3 ppm))	0.29	40-70 Hz
	Alkyl-methyl (0.90 ppm)	0.70	20 Hz
	α-Ester proton (4.15 ppm)*	N/A	30 Hz
ALC-0159	**Surface lipid proton**	**T_2 (s)**	**Peak width**
	PEG-methylene (3.71 ppm)	1.7	6 Hz
	PEG-methoxy (3.38 ppm)	3.3	4 Hz
	Methylene proton of N-alkyl chain (3.25 ppm)	0.12	21 Hz

Transverse (spin-spin, T_2) relaxation times of surface lipid proton in the LNP were measured using a CPMG (Carr Purcell Meiboom Gill)[1] based sequence with water suppression (excitation sculpting with gradients). The integrals were fitted and analyzed using Topspin 4.1. Relaxation measurements were collected by a Bruker NEO 600 MHz spectrometer, equipped with a 5 mm double resonance broad band $^{1}H/^{19}F$ (BBFO) cryoprobe at 25 °C (298 K). *No accurate T_2 value of α-Ester proton signal can be fitted due to the overlap impact from the residual sucrose signals.

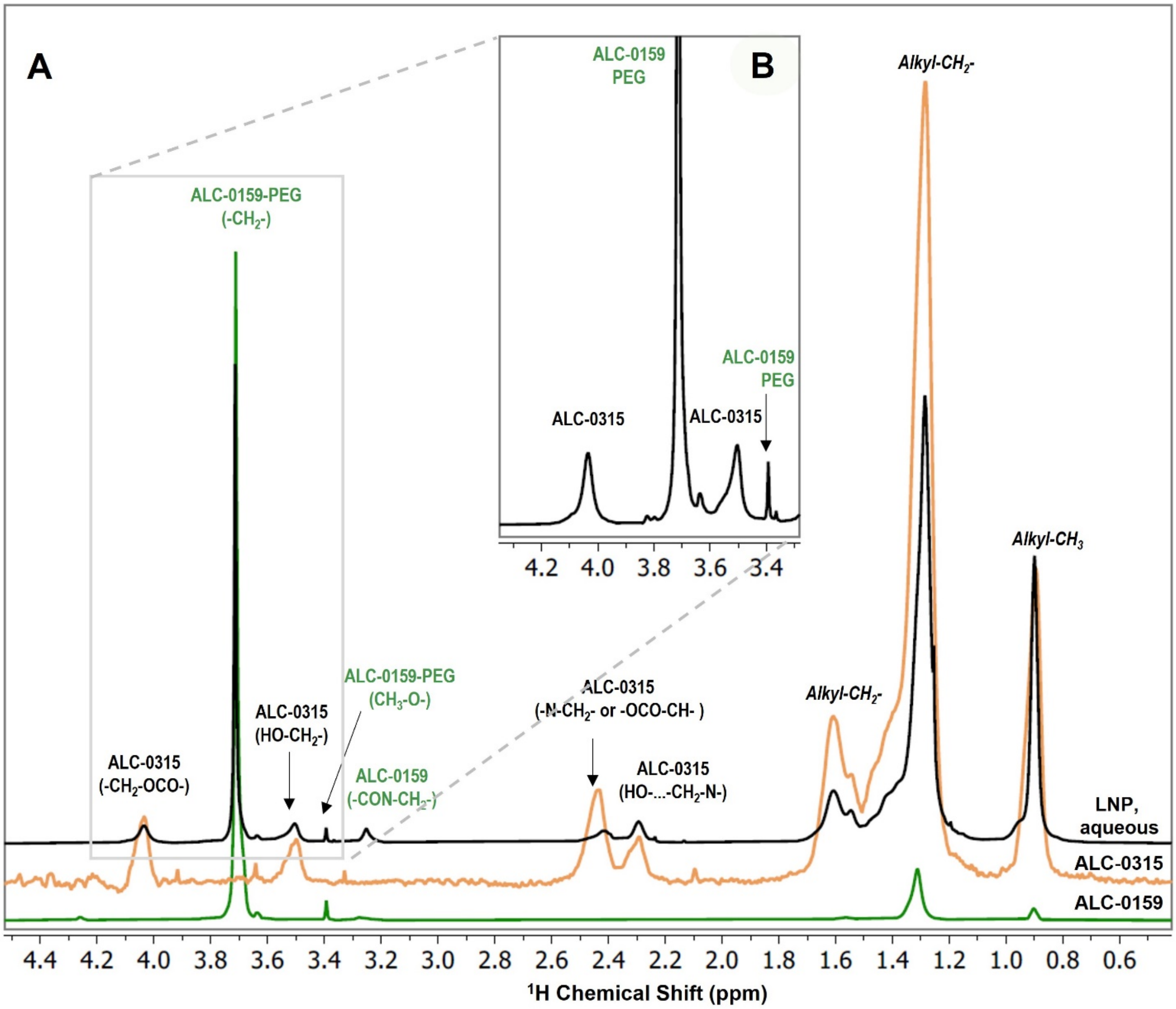

Figure S4. 1D ^{1}H NMR Overlay of Intact mRNA-LNPs, ALC-0159 and ALC-0315 at Absolute Abundance Ratio Determined by Disrupted LNP Analysis. A, top spectrum: intact mRNA-LNPs in aqueous phosphate buffer; Bottom two spectra: individual lipids: ALC-0315 (aqueous, PBS), ALC-0159 (aqueous, PBS). Proton signals in the mRNA-LNP spectrum were labeled in green for ALC-0159, and in black for ALC-0315; overlapped signals from both lipids were labeled in italic. The detected surface protons were annotated in the molecular structures of ALC-0159 and ALC-0315. B, an expansion of 4.2-3.0 ppm showing that in the intact LNP spectrum (black trace), the peak widths of ALC-0159-PEG (methylene and terminal methyl proton) signals are narrower than that of ALC-0315 peaks. In comparison of the LNP (black trace) and individual lipids in the aqueous environment (orange and green traces), the peak shapes are similar, because the individual lipids typically form micelles in the aqueous solution.

Determination of PEG Repeat Unit N Value in ALC-0159 PEGylated Lipid

ALC-0159 was dissolved in chloroform-d at 10 mg/mL. 1D ^{1}H spectrum was collected to determine the PEG repeat unit. Lipid NMR spectrum was collected by a Bruker NEO 600 MHz spectrometer, equipped with a 5 mm double resonance broadband $^{1}H/^{19}F$ (BBFO) cryoprobe at 25 °C (298 K). Spectrum was processed and analyzed using MestreNova 14.1.

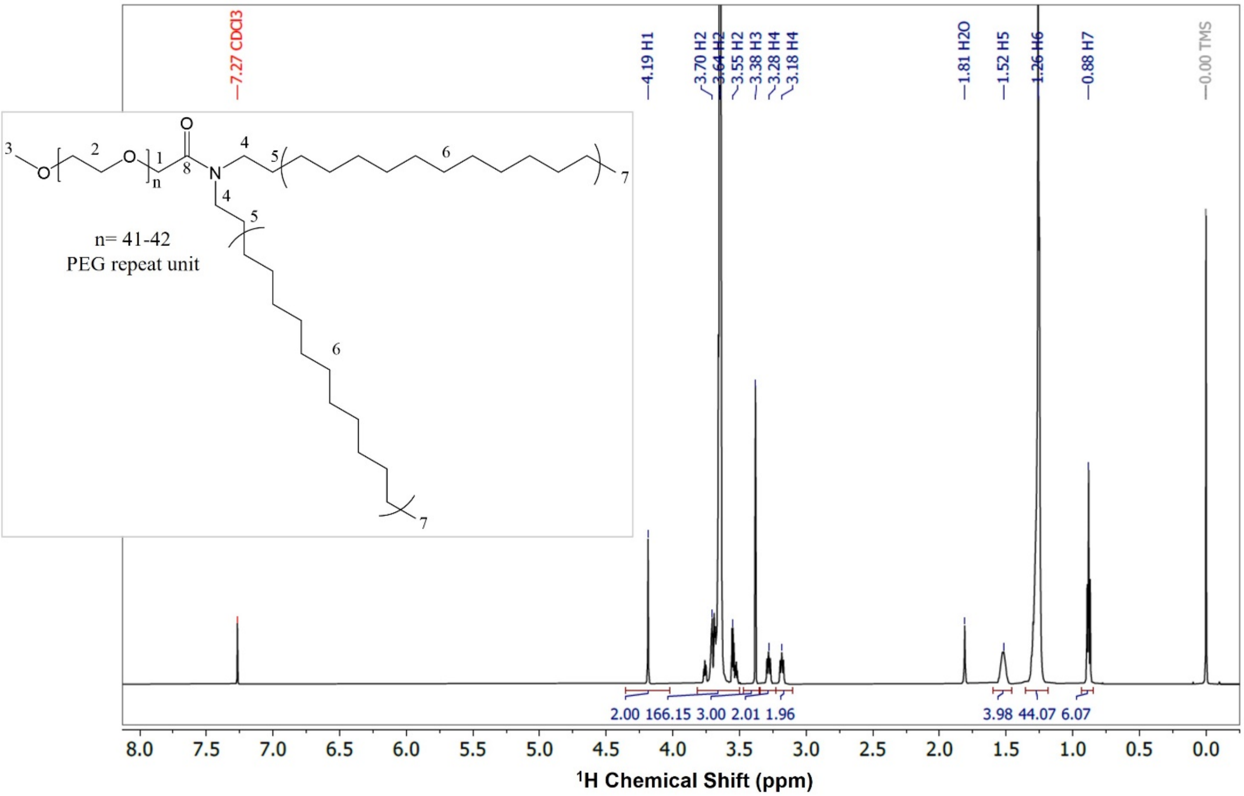

Figure S5. 1D ^{1}H Spectrum of PEGylated Lipid, ALC-0159 in Chloroform

Table S3. Proton Chemical Shift Assignments of ALC-0159 PEGylated Lipid

Number from Structure	Assignment	Chemical Shift (ppm)	Actual Protons	Theoretical Protons, PEG n=42
1	α protons on PEG chains	4.19	2.0	2
2	PEG methylene protons (terminus)	3.70, 3.55	166.2	168
2	PEG methylene protons (repeating unit)	3.64		
3	O-CH_3 (end of PEG chain)	3.38	3.0	3
4	Protons on C1 of N-alkyl chains	3.28	2.0	2
4	Protons on C1 of N-alkyl chains	3.18	2.0	2
5	Protons on C2 of N-alkyl chains	1.52	4.0	4
6	Methylene protons on N-alkyl chains	1.26	44.1	44
7	Terminal methyl protons on N-alkyl chains	0.88	6.1	6
		Total Protons:	229.2	231

Table S4. PEG Repeat Unit Characterization in ALC-0159 PEGylated Lipid

H Signals	H Type	Peak Region (ppm)	Peak Integral	H Count	N Value
1	CH_2	4.36 - 4.02	16580153	2	41.5
2	OCH_3	3.47 - 3.35	24871018	3	41.5
3	$2x(CH_2)$	3.34 - 3.11	32875668	4	41.9
4	$24x(CH_2)$	1.60 - 1.19	398295155	48	41.5
5	$2x(CH_3)$	0.93 - 0.85	50348953	6	41.0
Average N	$(CH_2CH_2)_n$	3.82 - 3.50	1377381489	4×N	41.5 ± 0.3

Particle Size Distribution and Morphology: Dynamic Light Scattering (DLS), Asymmetric Flow Field Flow Fractionation (AF4), and Cryogenic Electron Microscopy (Cryo-EM)

The particle diameter and polydispersity index were measured by DLS using Malvern Zetasizer Ultra. The analysis was performed at a scattering angle of 90° at a temperature of 25 °C using samples diluted in 0.1xPBS. The size distribution profile (data not shown) represents a representative batch of LNPs with a mean diameter of 77 nm and a narrow size distribution (polydispersity index <1).

The size distribution and morphology of the COVID-19 lipid nanoparticle (LNP) were characterized by Asymmetric Flow Field Flow Fractionation coupled to a multi-angle light scattering detector equipped with in-line dynamic light scattering capability (AF4-MALS-DLS) (data not shown) and Cryo-EM. AF4-MALS-DLS is using ASTRA software from Wyatt Technologies, Inc. The in-line DLS is used to determine hydrodynamic radius (Rh) and the MALS is used to determine the Root Mean Squares radius (Rz). The shape factor (Rz/Rh) was used to determine the shape or morphology of the LNP. A solid sphere should have a shape factor of 0.77, a hollow sphere a shape factor of 1.0, and more elongated molecules greater than 1. The weight average shape factor of the LNP as 0.86, which indicates the spherical conformation of the particle.

To prepare for cryo-EM imaging, LNP samples (4uL) were applied in triplicate to a 200-mesh lacey formvar coated gold grids (Electron Microscopy Sciences) and were vitrified using a ThermoFisher Vitrobot Mark IV system (at 5C with 100% humidity). Samples were imaged using a Talos F200C transmission electron microscope equipped with Ceta 4kx4k camera and an electron accelerating voltage of 200 kV. Images were taken at a magnification of 36,000x and 45,000x (1 second exposure time).

LNP Density Measurement

Lipid nanoparticle (LNP) density was experimentally determined using the density match analytical ultracentrifugation (AUC) method as described in Henrickson et.al.[2] LNP samples were diluted in formulation buffer with varying amounts of formulation buffer made with D_2O instead of water, resulting in varying buffer densities for each sample. A series of samples were made and SV- AUC experiments were performed. During analysis, only buffer viscosity was changed in the fitting algorithm, resulting in the movement of LNP species to lower s values. A plot of the movement of s values with buffer density gives a linear relationship (data not shown). By fitting a line to the data, and extrapolating to an s value of zero, the density of the particle was determined to be 1.0 g/mL.

Surface PEG Density Calculation

Detailed calculation protocol can be found in the previous work by Xu and co-worker.[3] The PEG density [Γ] (PEG molecules/100 nm^2) was determined by surface PEG moles (M_{PEG}, mole), total mass of nanoparticles (W_{NP}, g), the density of nanoparticles (d_{NP}, g/mL), and the particle diameter (D, nm) using Equation 1. The full surface coverage [Γ*] (unconstrained PEG molecules/ 100 nm^2) was determined by the molecular weight of the PEG chain (m, g/mol) using Equation 2 and 3. The ratio of [Γ/Γ*] is to assess the PEG conformation on the nanoparticle surface.

Equation 1 $$[\Gamma]=\frac{M_{PEG}\times 6.02\times 10^{23}}{W_{NP}/d_{NP}/\frac{4}{3}\pi\left(\frac{D}{2}\right)^3}\div 4\pi\left(\frac{D}{2}\right)^2\times 100$$

Equation 2 $\xi = 0.76m^{0.5}$ [Å]

Equation 3 $[\Gamma^*]=\frac{100}{\pi(\frac{\xi}{2})^2}$

Evaluation of the System Precision and the Method Precision for the NMR Method

A 1D ^1H diffusion experiment using Pulse Gradient Stimulated Echo (PGSTE)-bipolar gradients sequence was used for the LNP samples. The repeatability was evaluated by collecting triplicate measurements on a sample and preparing three different samples, respectively. LNP NMR spectra were recorded on a Bruker NEO 800 MHz spectrometer, equipped with a 5 mm proton-optimized triple resonance NMR inverse (TCI) cryoprobe at 25 °C (298 K). Spectra were processed and analyzed using MestreNova 14.1.

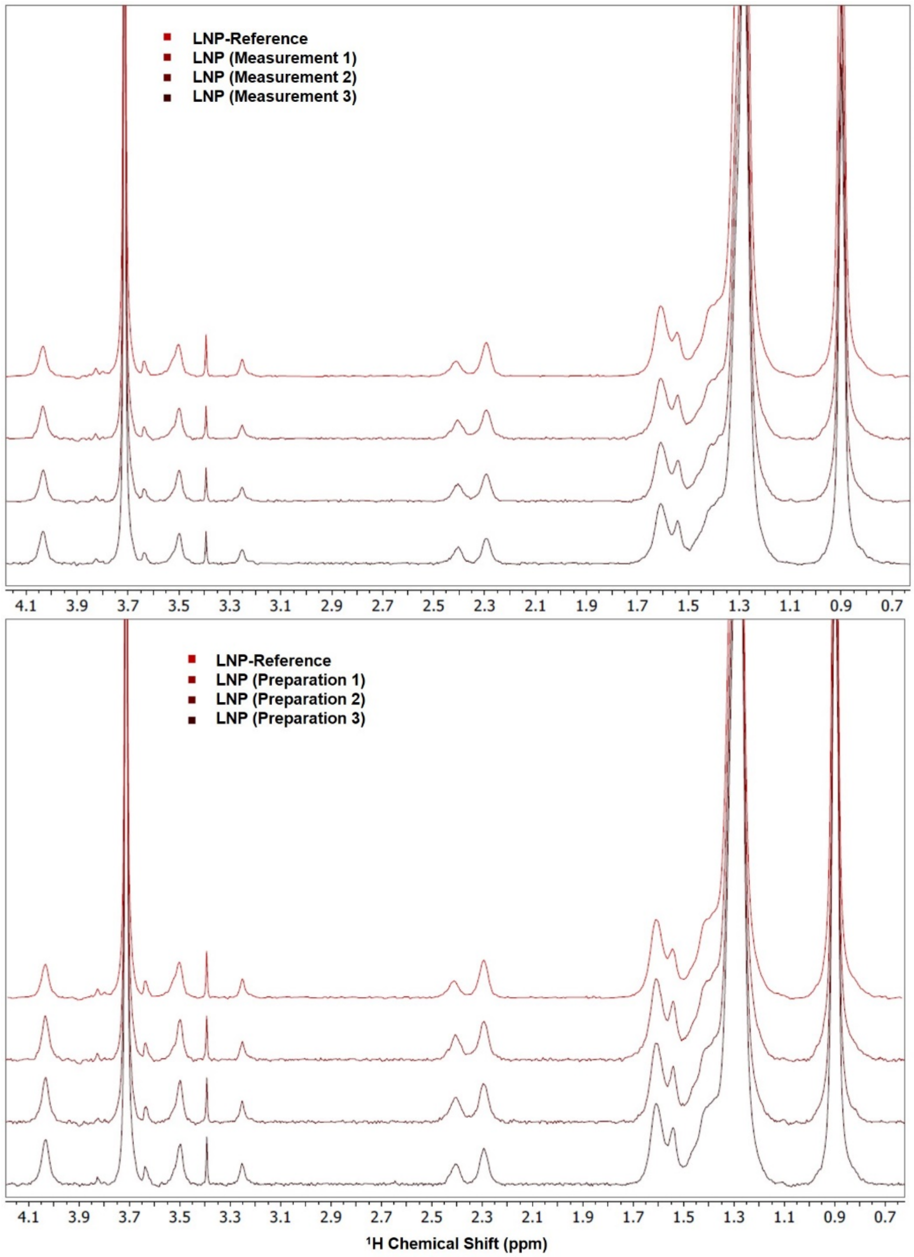

Figure S6. 1D ^{1}H PGSTE Spectra of Formulated LNP.

Supplementary 2D NMR for Assigning the Increased Signals in the Aged LNP

2D ^{13}C-^{1}H HSQC NMR spectra were recorded on a Bruker NEO 800 MHz spectrometer, equipped with a 5 mm proton-optimized triple resonance NMR inverse (TCI) cryoprobe at 25 °C (298 K). Spectra were processed and analyzed using Bruker TopSpin 4.1.4.

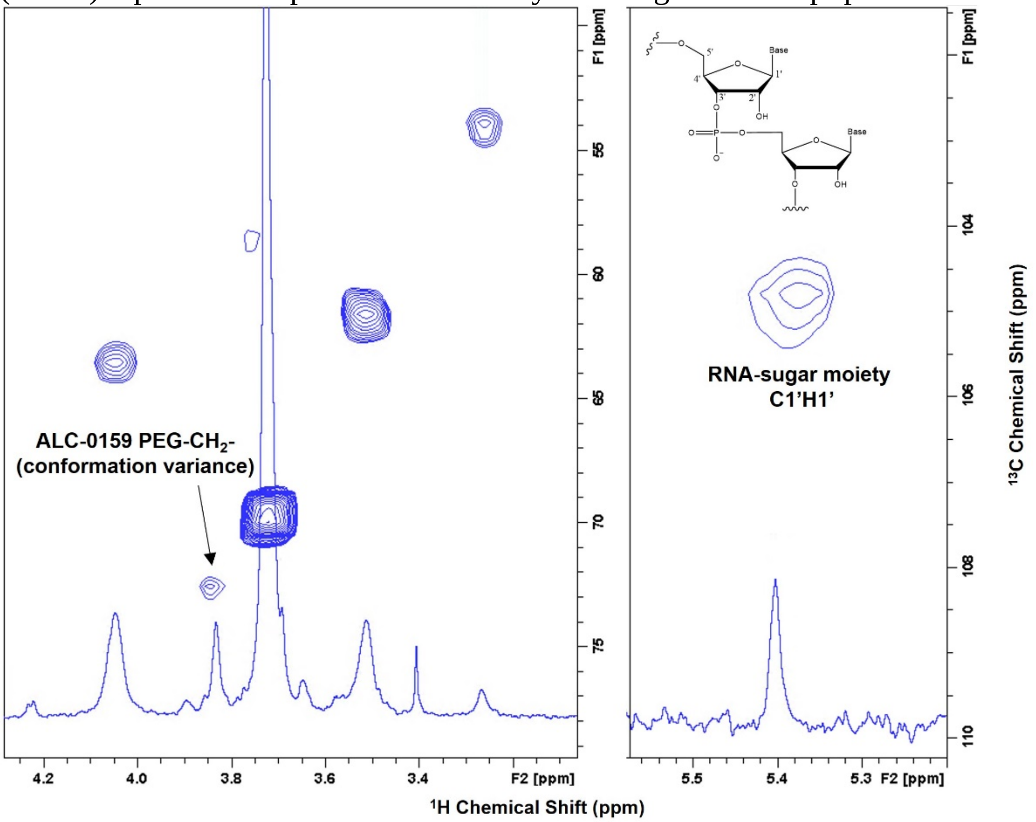

Figure S7. 2D ^{13}C-^{1}H HSQC Spectrum of a Representative Aged LNP.

Reference

1. Meiboom, S.; Gill, D., Modified Spin-Echo Method for Measuring Nuclear Relaxation Times. *Rev Sci Instrum* **1958,** *29* (8), 688-691.

2. Henrickson, A.; Kulkarni, J. A.; Zaifman, J.; Gorbet, G. E.; Cullis, P. R.; Demeler, B., Density Matching Multi-wavelength Analytical Ultracentrifugation to Measure Drug Loading of Lipid Nanoparticle Formulations. *Acs Nano* **2021,** *15* (3), 5068-5076.

3. Xu, Q. G.; Boylan, N. J.; Cai, S. T.; Miao, B.; Patel, H.; Hanes, J., Scalable method to produce biodegradable nanoparticles that rapidly penetrate human mucus. *J Control Release* **2013,** *170* (2), 279-286.
